# Analysis of operative efficacy for giant pituitary adenoma

**DOI:** 10.1186/1471-2482-14-59

**Published:** 2014-08-28

**Authors:** Shousen Wang, Shun’an Lin, Liangfeng Wei, Lin Zhao, Yinxing Huang

**Affiliations:** 1 Department of Neurosurgery, Fuzhou General Hospital, Fujian Medical University, No.156 Xihuanbei Road, Fuzhou 350025, P. R. China

**Keywords:** Giant pituitary adenoma, MR imaging, Tumor volume, Microsurgery, Transsphenoidal surgery, Operative efficacy, Resection rate

## Abstract

**Background:**

Surgical treatment of giant pituitary adenomas is difficult due to complicated dissection of the sellar area. The extent of tumor resection affects the efficacy of surgical treatment. This study is to investigate the efficacy of microsurgical treatment for giant pituitary adenoma and to analyze the relationship between treatment efficacy and tumor resection extent.

**Methods:**

A retrospective analysis was performed in 36 patients who received microsurgery to remove giant pituitary adenomas. The sizes of tumors before and after surgery were calculated with a novel method called the “platform-like volume calculation formula”. The relationships between extent of resection and the visual impairment recovery, and improvement of serum hormone level before and after operation were analyzed.

**Results:**

Two deaths were observed after surgery. And the gross and near-total resection was achieved in 8 cases, subtotal resection in 8 cases, mostly partial resection in 15 cases, and partial resection in 5 cases. The average resection rate was 72.8%. The resection rate of tumor with cavernous sinus invasion was significantly lower than those of patients without cavernous sinus invasion (P < 0.05). The improvement rate of hormone level in functional adenoma was 80.0%. Follow-up observations were carried out for 3 ~ 28 months in 25 cases. Visual improvement was observed in 64.0% of the cases.

**Conclusions:**

Microsurgical treatment can improve the visual impairment of the majority of cases and significantly decrease the serum hormone levels of functional adenoma. The extent of resection was significantly associated with cavernous sinus invasion.

## Background

Pituitary adenomas account for about 15% of primary intracranial tumors [1]. Pituitary adenomas with a diameter ≥ 4 cm are the giant adenomas, accounting for about 5% of the pituitary adenomas [2-5]. Because of complexity in dissection of the sellar area, surgical treatment of giant pituitary adenomas is difficult. According to the relationship between giant adenoma and cavernous sinus, Goel et al. divided pituitary adenomas into 4 grades and pointed out that the operation risk was high [3]. Some researchers used the endonasal endoscopic approach [5-7], the extended endoscopic endonasal transsphenoidal approach [8], the microscopic transsphenoidal approach [6], the extended pterional approach [3], the transsphenoidal-pterional approach [9], or the endoscopic transsphenoidal-ventricle approach [10-12] for tumor resection. However they did not accurately measure the extent of tumor removal.

This paper summarized 36 cases of giant pituitary adenomas treated in Fuzhou General Hospital. We applied the "platform-like volume calculation formula" to calculate the preoperative and postoperative tumor volume. The relationships between the extent of resection and operation approach, tumor size, visual acuity and hormone level were analyzed to evaluate the effect of microsurgical treatment for giant pituitary adenoma.

## Methods

### Enrollment criteria for giant pituitary adenoma patients

Inclusion criteria: 1) MRI examination showed lesions diameter ≥ 4 cm in sellar region; 2) Tumor resection was performed in Department of Neurosurgery, Fuzhou General Hospital, Fujian Medical University; 3) Pituitary adenoma was diagnosed by pathological examination. Exclusion criteria: 1) Pituitary adenoma resection was performed before admission; 2) History of preoperative radiotherapy.

### General information of enrolled giant pituitary adenoma patients

There were 36 patients enrolled in this study, including 22 males and 14 females. They aged from 20 to 76 years old, with an average age of 44 years. The course of disease ranged from 2 days to 11 years. Besides two coma patients, the other 34 patients all underwent ophthalmologic examination. Of them, 33 patients had decreased visual acuity with unilateral or bilateral temporal visual field defects and only 1 patient did not have vision or visual field obstruction. The detailed clinical data were shown in Table 1.

**Table 1:** Clinical features of 36 giant pituitary adenoma patients

**Clinical features**	**Number**	**Proportion (%)**
Decreased visual acuity^a^	33	97.1
Headache and dizziness	11	30.6
Coma	2	5.6
Dysendocrinia	17	47.2
Irregular menstruation	2	5.6
Acromegaly & irregular menstruation	1	2.8
Decreased sexual function	5	13.7
Amenorrhea	6	16.7
Hypothyrea	3	8.3

Note: ^a^Decreased visual acuity includes blindness, light sensitivity, finger counting and blurred vision.

Prior written and informed consent was obtained from every patient and the study was approved by the ethics review board of Fujian Medical University.

### Radiological data

Siemens 3.0 T superconducting magnetic resonance imaging (MRI) machine was used for coronal, axial and sagittal scanning of the sellar area. The scanning sequences were T1WI (TR / TE 400/10 ms) and T2WI (TR/TE 5000/120 ms) with thickness of 4 mm. The matrix was 250 × 250. The dynamic enhanced MRI used gradient echo (GRE) for T1WI enhanced scan. The maximum diameter of adenoma was 4.0 to 8.1 cm, with an average diameter of (5.38 ± 1.01) cm. The vertical height of adenoma was 2.9 to 7.5 cm, with an average height of (4.73 ± 0.95) cm. There were 14 cases of tumor with unilateral or bilateral cavernous sinus invasion (CSI) (Knosp grading ≥ 3), 3 cases of severe hydrocephalus, 9 cases of cystic tumor, 3 cases of apoplexy, and 2 cases with fluid-fluid level.

### Adenoma volume calculation

The platform-like volume calculation formula was used. To reduce the interference of sinus portion after enhancement on tumor volume calculation, coronal MRI was taken. Volume between two layers on MRI scan was considered a separate volume. The formula for volume calculation was v = [S_1_ + S_2_ + (S_1_ × S_2_)^1/2^] × h / 3. S_1_and S_2_ represent the upper and lower area respectively and h is the height. It has been shown mathematically that the arithmetic mean of the two numbers is greater than or equal to the geometric mean, which is the (S_1_ + S_2_) / 2 ≥ (S_1_ × S_2_)^1/2^. When S_1_ and S_2_ are infinitely close, (S_1_ + S_2_) / 2 will be infinitely close to (S_1_ × S_2_)^1/2^. For easy calculation, (S_1_ + S_2_) / 2 was used instead of (S_1_ × S_2_)^1/2^. Therefore a new formula for each platform volume calculation was used in this article, which was v = (S_1_ + S_2_ + (S_1_ + S_2_) / 2) × h / 3 = (S_1_ + S_2_) × h / 2. The tumor area of each layer on MRI scan was calculated by using Picture Archiving and Communication System (PACS) (Infinitt Healthcare Co., Ltd, Seoul, Korea), and then the total tumor volume was calculated by summing up the volume of each platform. Thus the final adenoma volume formula was V = (S_1_ + 2S_2_ + 2S_3_ + … + 2S_n ‒ 1_ + S_n_) × h / 2.

### Criteria for tumor resection extent

The extent of tumor resection was determined based on tumor volume before surgery and residual tumor volume after surgery. The percentage of residual tumor volume after surgery was defined as the ratio of residual tumor volume after surgery to tumor volume before surgery. Gross and near-total resection was defined when the percentage of residual tumor volume was less than 10%. Subtotal resection was defined when the percentage of residual tumor volume was between 10% and 20%. Mostly partial resection was defined when the percentage of residual tumor volume was between 20% and 40%. Partial resection was defined when the percentage of residual tumor volume was more than 40%.

### Tumor type

There were 26 patients with non-functioning pituitary adenomas. They showed clinical signs of decreased visual acuity, dizziness, headache, and decreased sexual function, etc. There were 7 patients with prolactin-secreting pituitary adenoma, whose preoperative serum prolactin levels were greater than 200 μg / L, and they chose surgery because of drug side effect intolerance or unsatisfactory with drug efficacy. There were 2 patients with thyrotropin-secreting pituitary adenomas, whose preoperative levels of TSH, free T3 and T4 in the serum were increased. There was 1 patient with growth hormone-secreting pituitary adenoma, showing as acromegaly. All specimens underwent immunohistochemical examination (Table 2).

**Table 2:** Endocrine diagnosis and immunohistochemical subtypes of patients (n = 36)

**Classification**	**Number**	**Proportion (%)**
**Clinical endocrine diagnosis**^**a**^		
Non-functioning adenoma	26	72.2
Prolactin-secreting adenoma	7	27.8
Thyrotropin-secreting adenoma	2	5.6
Growth hormone-secreting adenoma	1	2.8
**Immunohistochemical classification**		
Gonadotropin-secreting adenoma	14	38.9
Null cell adenomas	12	33.3
Prolactin-secreting adenoma	6	16.7
Thyrotropin-secreting adenoma	3	8.3
Multi hormone-secreting adenoma	1	2.8

Note: ^a^Clinical endocrine diagnosis was in accordance with WHO Classification, 2004 [12].

### Surgical procedure

Twenty-eight cases of patients underwent microscopic transsphenoidal surgery and 8 cases underwent microscopic pterional surgery. The pterional surgery generally chose the right approach. The left approach was chosen when the tumor was significantly bias toward the left side. Various structures were carefully dissected and tumor within the capsule was gradually removed under microscope, without damaging the surrounding structures. For the transsphenoidal surgery, supine position was used with neck hyperextension of 15° to 20°. And right nostril - septum - transsphenoidal approach was chosen to scrape as much tumor tissue as possible, until the diaphragma sellae satisfiedly sunk. Gelatin sponge and Otologic and Craniocerebral Glue (Guangzhou Baiyun Medical Adhdsive CO., LTD, Guangzhou, China) was used to seal off cerebrospinal fluid leakage.

### Statistical analysis

Data were presented as mean ± standard deviation (χ ± s), and analyzed by SPSS 19 statistical software (SPSS Inc., Chicago, Illinois, USA). P < 0.05 was considered as statistically significant.

## Results

### Tumor resection rate

To determine tumor resection rate, MRI scanning was conducted at 1 week before surgery and 1 d - 2 months after surgery. Tumor volume was calculated based on MRI radiological data. The significantly enhanced area in postcontrast T1WI scan was used as the calculation area. The average preoperative tumor volume was 40.5 ± 23.0 cm^3^, including volume of pituitary adenoma, normal pituitary tissue, cystic and stroke part. The average postoperative tumor volume was 11.5 ± 9.5 cm^3^. For tumor resection rate, there were 8 cases (22.2%, 8/36) of gross and near-total resection, 8 cases (22.2%, 8/36) of subtotal resection, 15 cases (41.7%, 15/36) of mostly partial resection, and 5 cases (13.9%, 5/36) of partial resection. And the overall resection rate was 72.8%.

Then the factors affecting tumor resection were analyzed (Table 3). Tumor resection rate in unilateral or bilateral CSI patients was significantly lower than those without CSI (Mann–Whitney U test, P = 0.018). There was no significant difference between surgical approach and resection rate (Mann–Whitney U test, P = 0.35). Preoperative tumor height and resection rate were not significantly correlated (r = 0.033, P = −0.848, Spearman test) while tumor volume and resection rate were not significantly correlated (r = −0.085, P = 0.623, Spearman test) (Data not shown). These results indicate that giant pituitary adenoma resection rate was associated with CSI, but not with surgical approach, tumor height or tumor volume.

**Table 3:** Comparison of resection rate between different groups (n = 34)

	**Number**	**Proportion (%)**	**Average tumor resection rate (%)**
Pituitary adenoma with CSI	14	41.2	62.0*
Pituitary adenoma without CSI	20	58.8	77.4
Transsphenoidal surgery	28	82.4	74.3
Craniotomy	6	17.6	66.8

Note: CSI, cavernous sinus invasion.

*P < 0.05, pituitary adenoma with CSI vs. pituitary adenoma without CSI, Mann–Whitney U test.

### Hormone level

To determine the effect of tumor resection on hormone levels, hormone levels were measured at 24 hours after resection. According to Mitsumori et al. [13], treatment efficacy with hormone levels returning to normal or reducing more than 25% is defined as improved. The cases with improved hormone level at 24 hours after surgery were counted, as shown in Table 4. As mentioned in the “Materials and Methods”, there were 10 cases of patients with functional pituitary adenoma. In the 7 patients with elevated preoperative prolactin, 5 patients (71.4%) showed improved hormone level at 24 hours after surgery. The remaining 3 cases of functional pituitary adenoma all showed improved hormone level at 24 hours after surgery. And the overall improvement rate was 80.0% (8/10). However, there was no significant difference in average tumor resection rate between patients with improved hormone level and patients with unimproved hormone level (Mann–Whitney U test, P = 0.506). This result shows that microsurgery could improve hormone level of most functional giant pituitary adenoma at 24 hours after surgery, and hormone improvement was not associated with tumor resection rate.

**Table 4:** Comparison of tumor resection rate with postoperative hormone (n = 10) and postoperative visual acuity (n = 24) improvement

**Improvement**	**Postoperative hormone**^**a**^		**Postoperative visual acuity**^**b**^	
	**Unimproved**	**Improved**	**Remained**	**Improved**
Number of cases	2	8	9	15
Average tumor resection rate (%)	72.3	83.5	60.4*	79.5

Note: ^a^Comparison of tumor resection rate in hormone levels in 10 patients with functional pituitary adenomas before and 24 hours after surgery. ^b^Comparison of tumor resection rate in visual acuity in 24 patients with decreased visual acuity before and 24 hours after surgery.

*P < 0.05, average tumor resection rate with remained visual acuity vs. average tumor resection rate with improved visual acuity, Mann–Whitney U test.

### Follow-up

There were 2 cases of craniotomy death at 9 d and 15 d respectively, which were non-functioning pituitary adenoma and thyrotropin pituitary adenoma respectively. Severe hydrocephalus was presented in both cases preoperatively. To treat the hydrocephalus, external ventricular drainage was applied in these two cases during surgery. And, cause of death was postoperative secondary intracranial hemorrhage and pulmonary infection respectively. Nine cases were lost to follow up and 25 cases were followed up at outpatient or by telephone, with follow-up time of 3 to 28 months. There were 3 cases who received second surgery. Among these 3 cases, 2 cases received two-stage surgeries because of relatively large tumor volume and 1 case undergone a second surgery because of residual tumor growth. There were 6 cases who received linear accelerator-based radiosurgery in our hospital and 1 case who received gamma knife radiosurgery in another hospital. The tumor control of these 25 patients was effective, without tumor growth or recurrence and no patient had refractory diabetes insipidus.

The preoperative headache and dizziness presented in 11 patients disappeared after surgery. Two patients without preoperative headache or dizziness complained occasional postoperative dizziness. There were 5 cases with preoperative sexual function declining, and the 2 cases who were followed up showed no improvement. Two patients remained irregular menstruation after surgery. Of the 6 cases with amenorrhea, 5 cases were followed up and remained amenorrhea. One preoperative conscious indifference patient showed significantly improved mental status. The Glasgow Outcome Scale (GOS) of these 25 cases of patients were shown in Table 5.

**Table 5:** Glasgow outcome scale (GOS) score of the follow-up patients (n = 25)

**GOS score**	**1**	**2**	**3**	**4**	**5**
Number	0	0	2	7	16
Rate (%)	0	0	8	28	64

The visual acuity was shown in Table 4. Among 25 cases with successful follow-up, 15 cases showed improved visual acuity with varied degrees and the average tumor resection rate of these 15 cases was 79.5%. Nine cases showed remained visual acuity and the average tumor resection rate of these 9 cases was 60.4%. The difference in average tumor resection rate between patients with remained visual acuity and patients with improved visual acuity was statistically significant (Mann–Whitney U test, P = 0.038). One patient with normal visual acuity before surgery, showed no visual acuity or vision disorders after surgery.

Prolactin-secreting pituitary adenoma patients took bromocriptine orally after discharge. The postoperative serum prolactin level in 1 case of unimproved patient and 4 cases of improved patients was higher than normal at 1 to 8 months after surgery. However the prolactin level in these patients after surgery decreased significantly compared with that before surgery. Hormone level of 1 thyrotropin-secreting pituitary adenoma patient returned to normal level at 3 months post surgery.

For non-functioning adenoma patients, 16 cases showed hypopituitarism before surgery, and received hormone replacement therapy after surgery. After treatment for 2 to 14 months, 14 patients were re-examined for hormone levels. Of them, 2 patients still had hypopituitarism and hormone levels in 12 cases returned to normal levels. Two patients with normal pituitary function showed normal hormone levels before and after surgery. The endocrine levels in the other patients were not examined.

### Representative cases

#### Case 1

**Figure 1:**
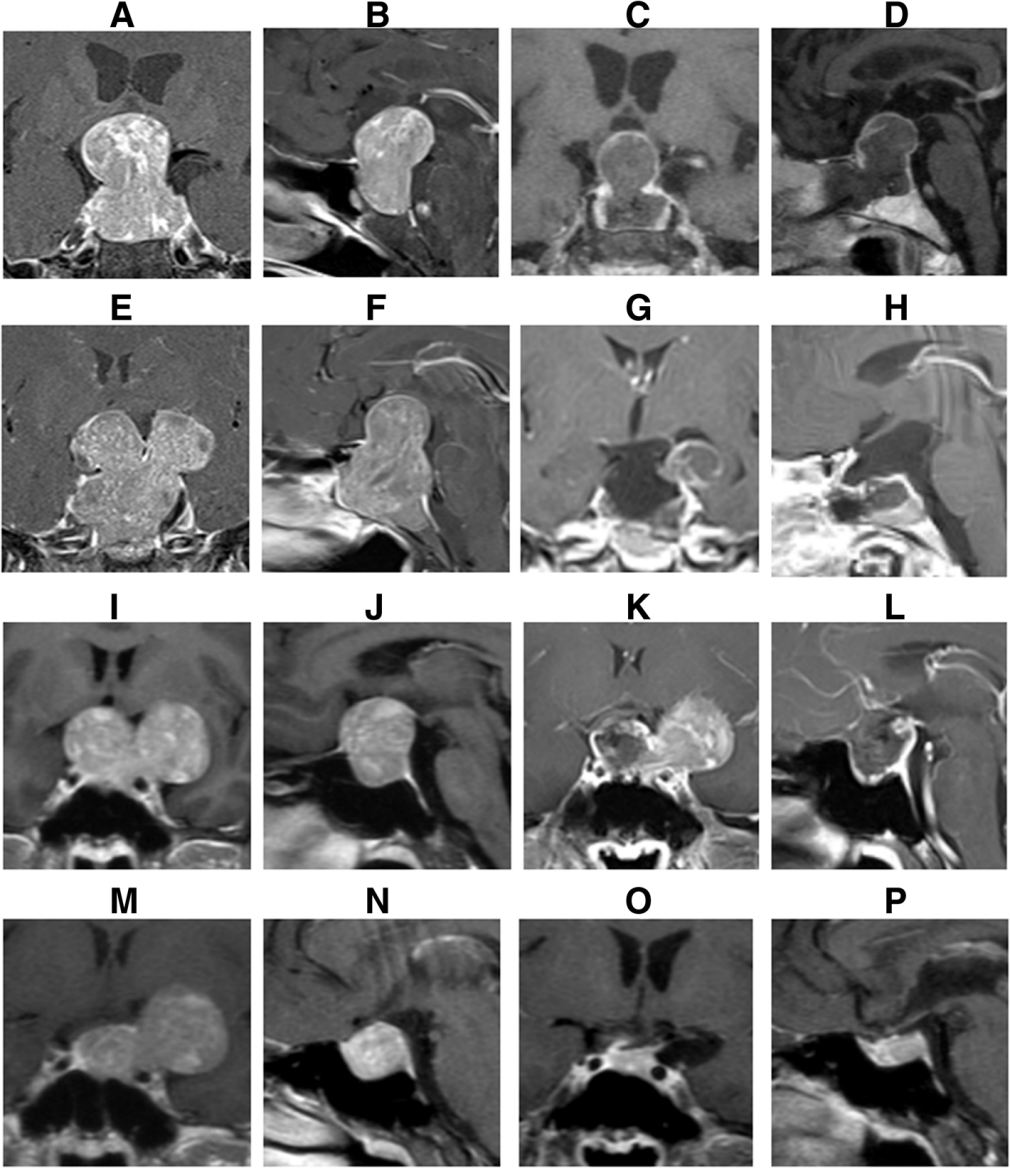
**MRI of representative cases before and after surgery. (A)** and **(B)** Coronal and sagittal MRI of Case 1 before surgery. **(C)** and **(D)** Coronal and sagittal MRI of Case 1 at 7 days after surgery. **(E)** and **(F)** Coronal and sagittal MRI of Case 2 before surgery. **(G)** and **(H)** Coronal and sagittal enhanced MRI of Case 2 at 2 days after surgery. **(I)** and **(J)** Coronal and sagittal MRI of Case 3 before surgery. **(K)** and **(L)** Coronal and sagittal enhanced MRI of case 3 at 2 days after surgery. **(M)** and **(N)** Coronal and sagittal MRI of Case 3 at 6 months after the first surgery. **(O)** and **(P)** Coronal and sagittal MRI of Case 3 at 1 year after the second surgery.

Male patient of 60 years old was admitted to hospital because of "recurrent visual impairment and visual obstruction for more than 5 years". The visual acuity of right eye was finger count/ 10 cm, and the left was 0.5. The serum level of follicle stimulating hormone (FSH) was 108.59 mIU/ml (normal: 1.4 to 18.1 mIU/ml), and the serum testosterone level was 144.04 μg/L (normal: 241 to 827 μg/l). Clinical endocrinology classification: non-functioning adenoma. MRI suggested space occupying lesion in sellar region (Figure 1A and B). Pituitary adenoma underwent transsphenoidal resection. Postoperative MRI suggested it was gross and near-total tumor resection (Figure 1C and D). Pathological classification: gonadotropin-secreting adenoma. The serum FSH level was 9.88 mIU/ml, and testosterone was 25.52 μg/l at 24 h after surgery. Postoperative binocular vision was improved.

#### Case 2

Female patient of 53 years old was admitted because of "blurred vision for two years and exacerbated for a month". Visual acuity of right eye was 0.3, and left eye was 0.5. Serum free T_4_ and estradiol level reduced. Clinical endocrinology classification: non-functioning adenoma. MRI suggested space occupying lesion in sellar region (Figure 1E and F). Pituitary adenoma was resected through transsphenoidal approach. Postoperative MRI suggested gross and near-total tumor resection (Figure 1G and H). Pathological classification: null cell adenoma. Serum levels of free T4, estradiol and cortisol decreased at 24 h after surgery. Hormone replacement therapy was received after discharge, and linear accelerator-based radiosurgery was performed at 4 months after surgery. At 11 months of follow-up, binocular vision was significantly improved, and serum hormone levels returned normal.

#### Case 3

Male patient of 33 years old was admitted because of "binocular vision loss with recurrent headache and dizziness for 1 year". Preoperative visual acuity of right eye was 0.5, and left eye was 0.4. Serum FSH level was 139.63 mIU/ml, and testosterone was 883.42 ng/dl. Clinical endocrinology classification: non-functioning adenoma. MRI suggested lesion in sellar region (Figure 1I and J). MRI results during the treatment of Case 3 were shown in Figure 1K-P. The pituitary adenoma was removed through left pterion approach and tumor invasion in the left cavernous sinus was observed during surgery. Pathological classification: gonadotropin adenoma. The serum FSH level was 67.84 mIU/ml and testosterone was 87.94 ng/dl 24 h after surgery. Six months after surgery, second craniotomy was performed. At 11 months of follow-up, visual acuity was improved.

## Discussion

Mortini et al. [14] showed that total resection rate of giant pituitary adenoma was 14.7%. Goel et al. [3] showed that the total resection rate was 29.65%, while Sinha et al. [15] showed a total resection rate of 74%. Koutourousiou [7] showed a near-total resection rate of 66.7%. The rate of gross and near-total tumor removal after transsphenoidal, transcranial, or combined procedures ranges from 14.7% to 74% in cases of giant pituitary adenomas, according to previously published reports [3,5,7,14,15]. In this study, the gross and near-total tumor resection rate was 22.2%, which was consistent with some of the previous studies. But it was different from Sinha’s results and Koutourousiou’s results. This inconsistence may be resulted from difference in surgical equipment, surgeon experience, tumor invasive extent and assessment methods.

The tumor diameter [16], or volume calculated by Tada formula [7,17] was considered as the main evaluation indicator for tumor size. However, tumor volume of pituitary adenoma calculated by Tada formula would generate great error because of the irregular shape and the multi-direction growth of pituitary adenoma. In this study, platform-like volume calculation formula was used. Tumor volume of each scanning layer was separately calculated and then the total tumor volume was obtained by summing up each separately calculated volume. Although each scanning layer was not a standard platform, the final result was more close to the actual volume compared with that calculated by Tada formula, particularly in tumor with irregular shape.

It is reported that compared to craniotomy, transsphenoidal surgery could reduce mortality and postoperative complications of pituitary adenoma patients [14,18]. Therefore transsphenoidal surgery was used in this study. Because of the tough texture of adenoma, it is more difficult to drop into sella during the operation, leading to resided upper tumor. Thus the texture has been considered an important factor for tumor resection [19]. Giant pituitary adenoma is highly invasive [20], often invading the cavernous sinus, internal carotid artery, hypothalamus and optic nerve. And the growth of adenomas was lobulated. All these characteristics lead to increased surgery risk and difficulty. For patient with preoperative CSI, the resection rate was significantly lower than those without CSI, which was consistent with previous studies [15,21].

Our results suggest that surgery could improve vision and hormone levels of most giant pituitary adenoma patients, and vision improvement was related with tumor resection rate. Additionally, surgery could significantly improve the symptoms of headache and dizziness. However surgery may not be effective for declined sexual function, irregular menstruation or amenorrhea.

Because of the small sample size, further research is needed. In this study, the visual acuity of patients before surgery was examined by professional ophthalmologists. Patients’ vision and visual fields were recorded. However, the visual acuity of patients after surgery was obtained by rough examinations of neurosurgeons and patients’ descriptions of their visual conditions. Compared with visual examinations by professional ophthalmologists, the data of patients’ visual acuity after surgery were more subjective and less objective. Further study is needed to obtain more objective visual acuity data of patients after surgery. Post operative MRI performed at different time points after surgery may affect the accuracy of tumor volume calculation. For example, MRI performed within the first post operative week may measure the volume of the hematoma while MRI performed after 2 months may measure the adenoma recurrences. In this study, because of the different postoperative MRI examination time, the measured postoperative tumor volume can not rule out the influence of hematoma and adenoma recurrence. Further studies with appropriate timing of the post operative MRI are needed.

## Conclusions

In summary, we found that surgery could improve hormone levels and visual acuity of most giant pituitary adenoma patients, and vision improvement was related with tumor resection rate. But hormone level was not related tumor resection rate. For sexual function decrease, irregular menstruation or amenorrhea, surgical treatment may not be effective. Tumor invasion to cavernous sinus would influence giant pituitary adenoma resection rate, while surgical tumor resection rate was not associated with surgical approach, tumor height or tumor volume.

## Abbreviations

MRI: Magnetic resonance imaging; GRE: Gradient echo; CSI: Cavernous sinus invasion; GOS: Glasgow Outcome Scale (GOS); FSH: Follicle stimulating hormone.

## Competing interests

The authors declare that they have no competing interests.

## Authors’ contributions

WSS and LSA performed the surgical procedure and drafted the manuscript. LSA, WLF, ZL and HYX participated in the design of the study and performed the statistical analysis. WSS conceived of the study, and participated in its design and coordination and helped to draft the manuscript. All authors read and approved the final manuscript.

## Pre-publication history

The pre-publication history for this paper can be accessed here:

http://www.biomedcentral.com/1471-2482/14/59/prepub
